# Implementation of Congestion-Related Controls Improves Runner Density, Flow Rate, Perceived Safety, and Satisfaction during an Australian Running Event

**DOI:** 10.3390/sports10090132

**Published:** 2022-08-31

**Authors:** Sean Peckover, Aldo Raineri, Aaron T. Scanlan

**Affiliations:** 1 School of Health, Medical and Applied Sciences, Central Queensland University, Rockhampton, QLD 4701, Australia; 2 School of Health, Medical and Applied Sciences, Central Queensland University, Brisbane, QLD 4000, Australia

**Keywords:** risk management, crowd dynamics, mass participation, marathon, crowd management, event tourism

## Abstract

This study examined the impact of congestion-related controls on runner density, flow rate, perceived safety, and satisfaction during an Australian running event. Runner congestion was compared between races organized at the Sunshine Coast Marathon and Running Festival in 2019 without controls and in 2021 with added controls, including modifications to the start corral design and use of wave starts. Following a mixed-method design, runner congestion was quantitatively measured via determining runner density and flow rate in the start corrals with video analyses, while post-event surveys were used to gather qualitative evidence regarding the prevalence of congestion and its impact on runner safety and satisfaction. Descriptive analyses for quantitative data showed runner density (1.48–3.01 vs. 0.52–1.20 runners per m^2^) and flow rate (102–152 vs. 36–59 runners per min per m) were reduced across races with controls. Regarding qualitative data, Wilcoxon–Mann–Whitney rank-sum tests demonstrated a significantly (*p* < 0.01) lower prevalence of congestion was perceived on course while running, alongside a reduced (*p* = 0.08) perceived impact of congestion on event satisfaction across races with controls. Furthermore, descriptive analyses for qualitative data showed a reduced proportion of runners indicated the start corrals were “somewhat” to “extremely” (rating of at least 3 on a 5-point scale) congested upon race commencement with controls (64% vs. 75%), and perceived safety (10% vs. 17%) and satisfaction (17% vs. 30%) were “somewhat” to “extremely” impacted by congestion across races with controls. Adopting suitable start corral designs with wave starts may enable race directors to reduce runner congestion to enhance continued participation among the public and viability of their running events.

## 1. Introduction

Mass participation running events contain large crowds that can encompass thousands of participants. These events can involve large portions of communities participating as runners, bringing potential positive impacts to public health [1]. However, the large participant base during running events can cause several undesirable consequences when the maximum number of persons for a designated space is exceeded, known as overcrowding [2]. Overcrowding, or runner congestion, during running events, can potentially cause injuries to participants via slips, falls, being trampled, and being crushed [2,3]. In this regard, survey data provided from 35 race directors with experience coordinating large running events in Australia indicated that almost half of the respondents (43%) had received feedback from runners related to congestion during their events, with this feedback mostly related to the impacts of congestion on runner satisfaction (94%) and safety (62%) [4]. Likewise, survey data from 222 runners who have participated in running events indicated most of them had experienced congestion when waiting for race commencement (93%), as the race commenced (97%), and on-course during the race (88%), with their experiences encountering congestion “somewhat” to “extremely” impacting (i.e., rating of at least 3 on a 5-point Likert item) their satisfaction (73%) and safety (43%) during the event [5]. In turn, many of the surveyed runners indicated they had tripped or slipped at the beginning of a race (38%), fell while running (27%), or required medical treatment (9%) due to congestion at running events [5], emphasizing participant congestion can lead to incidents and injuries of a concerning nature to race directors and runners alike. Furthermore, reduced participant satisfaction with a running event may negatively impact the reputation of the event among prospective consumers, which can jeopardize the financial viability of the event [5]. While race directors and runners have indicated congestion is a concern during running events, no data have been provided that precisely quantifies the congestion levels experienced by runners during large running events. 

Congestion levels are best determined via measuring the density and flow rate of runners participating in an event [2]. Density refers to the total number of persons occupying a designated area (i.e., persons per m^2^), while flow rate refers to the number of persons who pass an identified point across a designated period (i.e., persons per m per min) [2]. For application among pedestrians in using various community facilities, Fruin [6] identified objective density and flow rate values alongside their potential effects on pedestrian movement. More precisely, Fruin’s criteria stipulate six Levels of Service, where the first level corresponds to free-flowing crowds (density < 0.27 persons per m^2^ and/or flow rate < 23 persons per m per min) up to the sixth level indicative of critical situations where crowds have difficulty moving (density > 2.17 persons per m^2^ and/or flow rate > 82 persons per min per m) [6]. Still [2] describes the critical density point being reached when the flow of the moving crowd starts to reduce due to there being less free space resulting in person-to-person contact and potentially leading to crushing among the crowd. In this way, the risk of an adverse event occurring can be increased with either a higher crowd density or a higher flow rate [2]. For moving crowds specifically, guides have been developed stipulating the maximum number of persons that can be safely permitted within community spaces such as exits, walkways, and stadia [2]. For instance, the United Kingdom Guide to Safety at Sports Grounds sets a limit of four persons per m^2^ for moving crowds, with a flow rate capacity of 82 persons per m per min on level ground [7]. Likewise, Fruin [8] provided the following critical density levels for crowds in walking scenarios: 0.43 persons per m^2^, indicating crowd members can maintain normal walking speed while avoiding one another; 2 persons per m^2^, indicating crowd members reduce walking speed; 3.57 persons per m^2^ indicating crowd members experience involuntary contact with one another; and 5.55 persons per m^2^ indicating crowd members experience potentially dangerous forces. These guidelines have been used to interpret crowd dynamics among community members in various scenarios, such as attending religious events [9] and entertainment shows [10]. Such standards guide event organizers in effectively planning for crowd capacities at their event venues to allow for safe crowd access, participation, and egress within different spaces. While useful, these standards do not strictly cater to the management of running events, which are unique in that the crowd is unidirectional and aims to move as fast as possible over the set race distance, typically above walking speeds. 

While published congestion data are limited for participants during running events, Bain and Bartlo [11] provided foundation measurements from the 2017 Chicago Marathon. These authors reported an average runner density of 2.2 ± 0.1 persons per m^2^ in the start corrals, which is the designated area where runners wait to commence the race [11]. Although useful, Bain and Bartlo did not quantify the flow rate of runners in their investigation, nor did they provide congestion data as the race commenced. In this way, it is essential to quantify runner density and flow rate as the race commences, given this point is where runners mostly experience congestion and are exposed to the greatest crowd risk during running events [5]. Consequently, data describing the congestion experienced by participants during running events is currently lacking in the literature, making it unfeasible to identify the precise risk imposed on runners in these settings. 

When quantifying runner congestion during running events, it is important to consider the congestion-related controls implemented by race directors, as they each will likely impact the resultant runner density and flow rate data. In this regard, control options have been adopted in practice by race directors to mitigate congestion during running events, with most (74%) of the 35 race directors surveyed in a recent study indicating they have made changes to their event to reduce the impact of congestion [4]. More precisely, race directors indicated a preference to use wave starts (52%), limit the number of runners permitted to participate (43%), and change the course or start corral design (35%) as controls during the running events they have organized [4]. Similarly, a high proportion of the 222 runners surveyed in a separate study identified that seeding runners based on previous run times (91%), using wave starts (91%), and optimizing course design (89%) were effective ways to reduce congestion during running events [5]. Although the preferred controls to combat congestion during running events have been identified from race director [4] and runner [5] perspectives, the impacts of implementing suitable controls on runner congestion during running events have not been directly examined. Regarding the prominent controls preferred by race directors [4] and runners [5], the use of waves to facilitate crowd movement [12] and modification of physical design for exits (e.g., guiding system, width adjustment, buffer zones, funnel-shaped bottleneck) [13] have been regularly assessed for application in pedestrian scenarios, but not running events. 

Research identifying the efficacy of control strategies to reduce congestion during running events may provide evidence that will benefit participants by reducing their risk of experiencing negative incidents associated with overcrowding and in turn enhance their satisfaction with the event to strengthen their intention to participate in that event into the future [14]. In turn, continued participation in running events may increase physical activity and sports participation within the community [15], bringing potential positive impacts on public health [16]. Moreover, race directors may also benefit from this research through reducing their litigation risk from participants who encounter congestion-related incidents and enhancing the experience of runners to improve the reputation of the event to promote continued participation, enhancing its financial viability in the process [17]. Therefore, the aims of this study were to quantify runner congestion (density and flow rate) upon race commencement in the start corrals and examine the impact of controls on the density, flow rate, perceived safety, and perceived satisfaction of runners during an Australian running event. 

## 2. Materials and Methods

### 2.1. Experimental Design

A mixed-method design was adopted in this study. More precisely, quantitative methods involving video analyses were used to measure the density and flow rate of runners in the start corrals upon commencing different races at a large Australian running event. Quantitative analyses were restricted to the start corrals at each running event (rather than at different segments of the course) as this is where runners have previously indicated they experience the most congestion [5]. In turn, qualitative analyses of the same running event were conducted using electronic surveys to measure runner perceptions on congestion prevalence, as well as the subsequent impact of congestion on their safety and satisfaction. To recruit a suitable running event, race directors organizing large Australian running events were approached to take part in the study. Accordingly, the Sunshine Coast Marathon and Community Run Festival was monitored in this study on two occasions, firstly in 2019 without controls and secondly in 2021 with added controls. At each of these events, three separate race groupings were administered, including the (1) Marathon and Half-Marathon, (2) 10-km race, and (3) 5-km race. The race director was provided with the research aim and methodology, before providing consent for data collection to occur at their event. All procedures in this study were approved by the Central Queensland University Human Research Ethics Committee (approval number: 0000020995).

### 2.2. Quantitative Procedures

#### 2.2.1. Measurement of Runner Density

The process adopted to measure runner density in the start corrals during large running events was established from previous methodology applied in another large running event [8]. Specifically, four plastic telescopic poles (each 2.2 m high) were affixed onto crowd control barriers in the start corrals and had reflective tape attached to their top ends to enable visual identification of each pole during video analysis. The poles were positioned ~2.0–3.0 m apart on the crowd barriers around the start corral to form a quadrilateral-shaped area in which runners were manually counted using video footage. The course design and start locations were identical across the 2019 and 2021 events, with the Marathon and Half-Marathon using a different start location to the 10-km and 5-km races. Accordingly, the designated space to quantitatively measure runner density differed between races (i.e., Marathon and Half-Marathon vs. 10-km and 5-km races) and between events due to slight variations in crowd barrier placement. All distance measurements were taken using an electronic laser device (GLM 80 Professional, BOSCH; Stuttgart, Germany) to calculate the area (m^2^) of the designated space for determining runner density using Bretschneider’s formula (2019 event—Marathon and Half-Marathon = 29.10 m^2^, 10-km and 5-km races = 12.95 m^2^; 2021 event—Marathon and Half-Marathon = 20.04 m^2^, 10-km and 5-km races = 17.21 m^2^). A video camera (Go-Pro Hero 6, Woodman Labs Inc.; Half Moon Bay, CA, USA) was affixed to the top of a separate telescopic pole at a height of ~3.5 m to capture video footage of runners as the race commenced. This height was chosen to provide a suitable angle to capture all runners located in the identified area at any point in time. Video footage was recorded ~5 min prior to the start of each event until the last runner passed the start line following race commencement. After each event, video footage was downloaded from the camera and transferred to a computer for analysis using Windows Media Player (version 12, Microsoft Corporation; Redmond, WA, USA). The captured footage was reviewed on a large screen (~140 cm) in ultra-high definition, with the identified area for counting runners drawn onto a transparent plastic sheet for ease of recognition. An example image with a count of runners taken from the video analyses is shown in Supplementary Figure S1. Video analyses were used to manually count the number of runners in the identified area 1 min prior to and each minute following the commencement of the race. The total number of runners identified in the frame within the designated space was counted and then divided by the area to calculate the density of runners at that point in time. All video analyses were performed by a single researcher. Consequently, to assess the intra-rater reliability of this approach, the same researcher performed two analyses of each race at each event separated by 7 days to avoid data recall. In turn, the retest reliability of manually determining the number of runners in the designated area across all races and events was deemed acceptable (coefficient of variation = 1.1–2.3%; intraclass correlation coefficient = 1.00) [18]. 

#### 2.2.2. Measurement of Runner Flow Rate 

To calculate runner flow rate, the width of the start line timing mat in each event was measured using an electronic laser device (GLM 80 Professional, BOSCH; Stuttgart, Germany). The timing mat width was different between races within each event due to the varied start locations adopted and slightly differed across events due to variations in crowd barrier placement. Accordingly, the start line timing mat widths were measured as: 2019 event—Marathon and Half-Marathon = 7.70 m, 10-km, and 5-km races = 7.30 m; 2021 event—Marathon and Half-Marathon = 7.55 m, 10-km and 5-km races = 5.55 m). During all events, runners were required to wear a race bib containing a radio frequency identification (RFID) chip to identify when they crossed the start line timing mat and calculate race splits and finish times. Following each event, the electronic timing data from each runner was accessed in an anonymized form with the runner code and time (hh:mm:ss.SSS) that they crossed the start line. These data were inputted into a spreadsheet (Microsoft Excel; version 2205, Microsoft Corporation; Redmond, WA, USA) and then used to calculate the number of runners crossing the timing mat across each minute from the commencement of the race (i.e., summing the runners crossing the mat to calculate the total number of runners in each minute). In turn, the number of runners crossing the timing mat was divided by the width of the timing mat to calculate the flow rate as runners per m per min. 

### 2.3. Qualitative Procedures

An online survey was used to determine the perceptions of runners regarding their experiences with congestion during each event examined in this study. An online survey was chosen for qualitative assessment given it is a low-cost and efficient method to collect data compared to face-to-face interviews, mail surveys, or telephone interviews [19,20] and has been regularly adopted to examine perceptual responses in runners following running events [9,21]. In this regard, a survey instrument was adapted from previous research [5]. The original survey underwent multiple phases of face validation to assess the perspectives of runners regarding the prevalence and impact of congestion during running events [5]. However, for this study, the original survey was adapted for administration to runners who had completed the specific event and did not ask about their general experience with race congestion. Questions were added to the start of the questionnaire regarding the race length the participant had completed and if they had previously participated in the event. Questions adopted in the original survey related to common controls used to reduce congestion during running events were removed in this study as they were not relevant. The final survey questions distributed to runners are shown in Table 1, which included dichotomous yes/no, multiple-choice, Likert-type, and open-ended questions. The survey questions were designed for ease of completion on mobile devices [19]. The final question of the survey was a free-text question, with coded analyses of keywords and phrases conducted to identify themes in responses. 

Details regarding the purpose, significance, and risks of participation in the research, as well as the anonymity of results, were made clear to survey participants via an information notice presented at the commencement of the online survey. Personal data such as age, sex, or ethnicity were not collected to protect the anonymity of participants. Before commencing the online survey, participants were notified they could leave the survey at any stage if they did not wish to participate further. Data from participants leaving the survey without completing it entirely were not retained in the study. 

Surveys implemented for the 2019 event were hosted using SurveyMonkey (Momentive Inc.; San Mateo, CA, USA), while surveys implemented for the 2021 event were hosted using Qualtrics (Qualtrics; Provo, UT, USA). Once finalized, the survey was distributed by the race directors following each event either via direct email to runners that contained a link to the survey (2019 event) or via a Facebook post that contained the link (2021 event). Participants were restricted to one survey completion per internet provider (IP) address to prevent multiple survey completions from the same participant. The survey remained open for four weeks after the conclusion of each event.

### 2.4. Congestion Controls Implemented 

The controls implemented in each of the examined running events are shown in Table 2. While each event included controls to manage runner congestion, comparisons in quantitative and qualitative data were made between events adopting different controls to identify the potential impact they had on runner congestion. In this way, a direct comparison in runner congestion could be made between the 2019 and 2021 events, given the same course design and similar number of runners participating across events, including 4890, in 2019 (2550 in the Marathon and Half-Marathon, 1430 in the 10-km race, and 910 in the 5-km race) and 4235 in 2021 (2621 in the Marathon and Half-Marathon, 1081 in the 10-km race, and 533 in the 5-km race).

**Figure 1:**
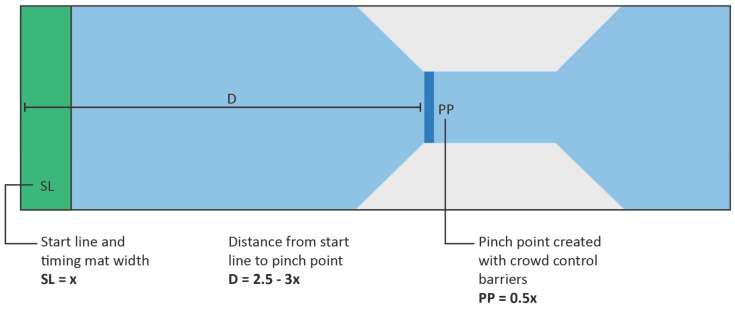
Modified start corral design implemented at the 2021 Sunshine Coast Marathon and Running Festival*. Note*: SL = starting line; D = distance; PP = pinch point.

Two major controls were added to the 2021 event, including modification of the start corral design and the use of wave starts during each race. Regarding start corral design, a standard start corral has the same width as the timing mat, whereby runners enter the corral and wait for the race to commence before transitioning from a stationary position to running. The modified corral design (Figure 1) was designed to control the flow of runners onto the course with the use of a pinch point with funneled ends to filter the runners onto the course in a more controlled manner. Furthermore, the pinch point in the modified design was a sufficient distance from the timing mat so that runners were able to pass through it at a walking pace before commencing running. Sun et al. [22,23] documented that the use of a funnel end at the egress point following a pinch point or bottleneck design increases movement velocity and reduces stopping motions among crowds. This design is beneficial to runners as they can accelerate from a walk to a running pace prior to the start of the race as they cross the start line timing mat. The use of a pinch point also assisted race organizers in stopping the flow of runners when commencing each subsequent wave.

The use of wave starts involved an initial elite group of runners identified by the race organizer, followed by subsequent waves of runners being released in a controlled manner. Each wave of runners following the elite group was released from behind the pinch point in the start corral. Runners (all except the elite group) self-selected the start wave that aligned with their goal race time, where faster runners were placed closer to the start. The starting times for each wave in each race were set by the race organizer and communicated via the public address system at the race starting area. 

### 2.5. Statistical Analyses

Quantitative data (runner density and flow rate) are presented as single data points for each minute during each race in each event. Descriptive comparisons in quantitative data were made between events given the limited number of independent running events able to be examined and discrete timepoints used for analysis in each event. Qualitative data (survey responses) are presented as the number and percentage of respondents selecting each response for each question at each event. Given survey data collected from each event was mostly independent (i.e., only some participants may have completed the survey following both events) and categorical, Pearson’s Chi-square test (χ^2^) was used to compare the distribution in survey responses for each question (except questions 2 and 10) between the 2019 and 2021 events [23]. Moreover, while various approaches have been advocated for analyzing survey questions using Likert scales [24], medians and interquartile ranges were calculated for Likert items in our survey (questions 5 to 9) alongside Wilcoxon–Mann–Whitney rank-sum tests for comparing outcomes between the 2019 and 2021 events [25]. Data were processed using Microsoft Excel (version 2205, Microsoft Corporation; Redmond, WA, USA), with statistical analyses performed using JASP (version 0.16.3, JASP Team; Amsterdam, Netherlands). Statistical significance was accepted when *p* < 0.05.

## 3. Results

### 3.1. Quantitative Analyses

**Figure 2:**
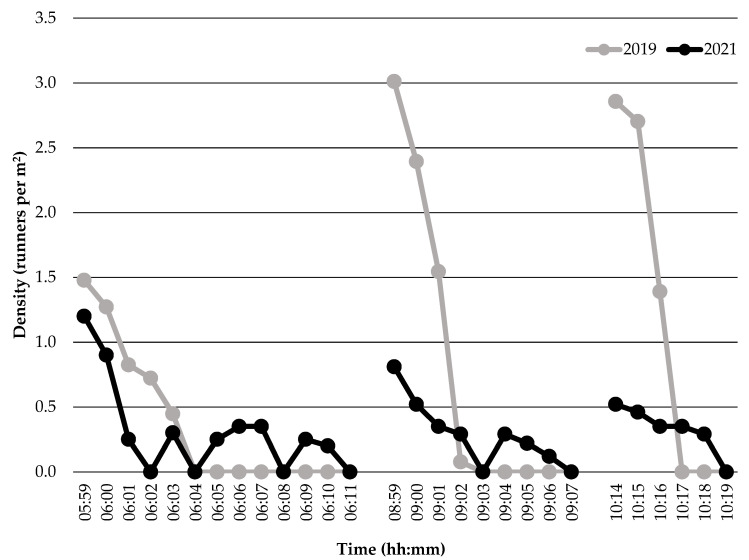
Runner density during the Sunshine Coast Marathon and Running Festival events held in 2019 without controls and 2021 with controls. *Note*: Start times in 2019 were: 06:00 for the Marathon and Half-Marathon; 09:00 for the 10-km race; and 10:15 for the 5-km race. Start times in 2021 were: 06:00 (elite wave), 06:01 (wave 1), 06:03 (wave 2), 06:05 (wave 3), and 06:09 (wave 4) for the Marathon and Half-Marathon; 09:00 (elite wave), 09:01 (wave 1), 09:02 (wave 2), and 09:03 (wave 3) for the 10-km race; and 10:15 (elite wave), 10:15:30 (wave 1), and 10:17 (wave 2) for the 5-km race.

Runner density data collected from the Sunshine Coast Marathon and Running Festival events in 2019 and 2021 are shown in Figure 2. Comparisons in the descriptive data show higher runner densities were apparent during the 2019 event compared to the 2021 event (with added controls) 1 min prior to the race start, upon race commencement, and 1 min following race commencement across all races (Marathon and Half-Marathon, 10-km race, and 5-km race). Moreover, peak runner density was 23% higher during the Marathon and Half-Marathon start (1.48 vs. 1.20 runners per m^2^), 271% higher during the 10-km race start (3.01 vs. 0.81 runners per m^2^), and 450% higher during the 5-km race start (2.86 vs. 0.52 runners per m^2^) in 2019 compared to 2021.

**Figure 3:**
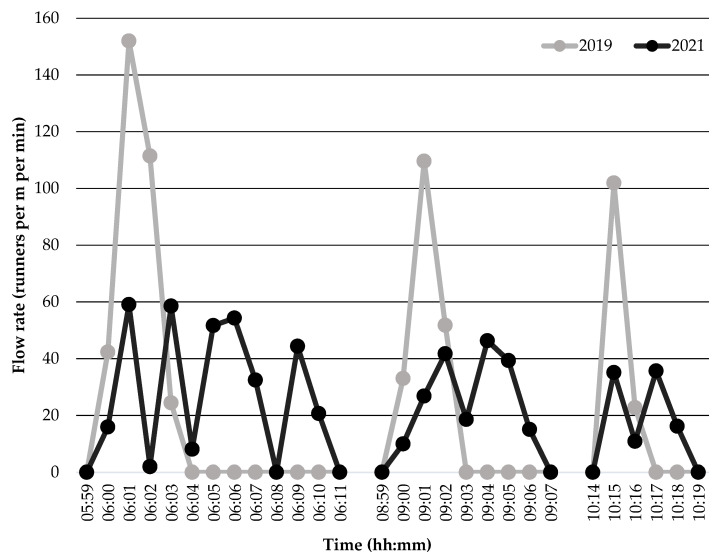
Runner flow rate during the Sunshine Coast Marathon and Running Festival events held in 2019 without controls and 2021 with controls. *Note*: Start times in 2019 were: 06:00 for the Marathon and Half-Marathon; 09:00 for the 10-km race; and 10:15 for the 5-km race. Start times in 2021 were: 06:00 (elite wave), 06:01 (wave 1), 06:03 (wave 2), 06:05 (wave 3), and 06:09 (wave 4) for the Marathon and Half-Marathon; 09:00 (elite wave), 09:01 (wave 1), 09:02 (wave 2), and 09:03 (wave 3) for the 10-km race; and 10:15 (elite wave), 10:15:30 (wave 1), and 10:17 (wave 2) for the 5-km race.

Runner flow rate data collected from the Sunshine Coast Marathon and Running Festival events in 2019 and 2021 are shown in Figure 3. Comparisons in the descriptive data revealed higher runner flow rates during the 2019 event compared to the 2021 event upon race commencement and 1 min following race commencement across all races. In addition, peak runner flow rate was 157% higher during the Marathon and Half-Marathon start (152 vs. 59 runners per min per m), 112% higher during the 10-km race start (110 vs. 52 runners per min per m), and 183% higher during the 5-km race start (102 vs. 36 runners per min per m) in 2019 compared to 2021.

### 3.2. Qualitative Analyses 

Overall, there were 40 responses to the survey in 2019 and 172 responses to the survey in 2021 from runners participating in Sunshine Coast Marathon and Community Run Festival. Characteristics of survey respondents are contained in Table 3. No significant differences in the distribution of responses were apparent between the 2019 and 2021 events. Most respondents participated in the Marathon or Half-Marathon (80% in 2019 and 75% in 2021) and were serious runners (70% in 2019 and 69% in 2021) with at least 3 years of running experience (68% in 2019 and 67% in 2021).

Responses to survey questions related to the prevalence and impact of congestion during the 2019 and 2021 events are presented in Table 4. Wilcoxon–Mann–Whitney rank-sum analyses revealed a significantly (*p* < 0.01) greater perceived prevalence of congestion on course while running in the 2019 event compared to the 2021 event. Similarly, the greater perceived impact of congestion on event satisfaction in the 2019 event compared to the 2021 event was approaching significance (*p* = 0.08). Non-significant variations in responses were evident between events for all remaining questions. Further, descriptive analyses regarding the proportion of participants experiencing congestion and the impacts of congestion revealed some notable trends. Specifically, a greater proportion of participants indicated they “somewhat” to “extremely” experienced congestion (i.e., rating of at least 3 on a 5-point Likert item) in the 2019 event prior to race commencement (75% vs. 65%), upon race commencement (75% vs. 64%), and on course while running (30% vs. 17%) compared to the 2021 event. In turn, a greater proportion of participants indicated that congestion “somewhat” to “extremely” impacted their safety (17% vs. 10%) and satisfaction (30% vs. 17%) during the 2019 event than in 2021. Regarding incidents experienced at each event, a higher proportion of participants witnessed or experienced crushing or pushing while waiting to run (18% vs. 10%) and tripping or slipping at the beginning of races (10% vs. 2%) in 2019 compared to 2021.

## 4. Discussion

This study examined the impact of congestion-related controls on the density, flow rate, perceived safety, and perceived satisfaction of runners within the start corrals during an Australian running event. In this regard, the effects of a multifaceted congested-related control approach involving changes in the start corral design and use of wave starts on quantitative (runner density and flow rate) and qualitative (perceived prevalence, safety, and satisfaction) measures of runner congestion were examined during the Sunshine Coast Marathon and Running Festival in 2019 without controls and in 2021 with added controls. Although performed in a real-world context, the comparison was well controlled with consistent participant numbers alongside identical start locations, start times, and course designs being used across events. Furthermore, almost half (49%) of runners surveyed at the 2021 event participated at the 2019 event. In this way, the outcomes provide ecologically valid evidence of the potential benefits of using control strategies to combat runner congestion in the start corrals, as this point of the race is a prominent source of congestion during running events [5].

Runner density is a pertinent measure to consider when quantifying runner congestion during running events, as it has been deemed a necessity to assess the complete criticality of a situation [26]. Results of this study showed peak runner density was ~3 runners per m^2^ in the 10-km and 5-km races during the 2019 event without the added control strategies. These values fit the highest level (out of six levels) within Fruin’s Level of Service model (>2.17 people per m^2^) [6], suggesting critical density levels were apparent, increasing the likelihood of runners having to stop and subsequently contacting others. In contrast, peak runner density was between 0.5–1.2 runners per m^2^ across races in the 2021 event, fitting the third (0.43–0.72 people per m^2^) and fourth levels (0.72–1.08 people per m^2^) within Fruin’s Level of Service model [6], indicating runners were less crowded upon starting races. Comparisons in runner density patterns in the start corrals upon race commencement (Figure 2) across events suggest the reduction in runner density could be attributed to the implemented control strategies. Regarding the modified corral design, Still [27] identified crowd queuing systems that reduce pedestrian density are necessary to limit instances of crowd surges, slips, trips, falls, and crowd collapse. In this way, the adapted start corral design included a pinch point with funnel endings prior to the start line, which simultaneously restricted the number of queued runners moving forward in the start corrals to commence the race at any one time and subsequently provided a large space for runners exiting the pinch point to filter into. These mechanisms reduced runner density upon race commencement by limiting the number of runners able to cross the start line simultaneously. Furthermore, the use of wave starts for each race in the 2021 event likely explain the more consistent lower peaks in runner density compared to the single high peak in the 2019 event (Figure 2). Using wave starts limited the number of runners vying to start each race at the same time, which dispersed runners more evenly across time in the start corrals as opposed to promoting a large dense single crowd with a single release of runners. The use of wave starts also likely had a synergistic effect on runner density alongside the start corral design, given pinch points can become ineffective when the inflow of people exceeds its capacity [28]. Consequently, the wave releases effectively controlled the inflow of runners entering the pinch point. These findings suggest the combined use of a modified start corral design and wave starts may lower runner density in a dynamic crowd scenario at the start of running events, reducing the potential safety risks to participants [27]. 

It is essential to consider runner flow rate alongside runner density when assessing runner congestion in the start corrals at running events. Examining running events is unique from other scenarios as the crowd is unidirectional and competitive in nature [27]. Results of this study showed peak runner flow rate across races in the 2019 event without controls ranged from 102–152 persons per m per min. These peak runner flow rates fit the highest level (out of six levels) within Fruin’s Level of Service model (>82 persons per m per min [6], suggesting critical flow rates were experienced. In contrast, substantially lower peak runner flow rates (<60 runners per m per min) were apparent across races in the 2021 event with controls, fitting the third (33–49 persons per m per min) and fourth levels (49–66 persons per m per min) within Fruin’s Level of Service model. The varied patterns in runner flow rate between the 2019 and 2021 events (Figure 3) suggest the control strategies likely had a positive impact on the crowd dynamics and congestion within the start corrals. Specifically, the use of wave starts creates a hold and release system [27], allowing the static crowd waiting in the start corrals to transition into a dynamic crowd in a controlled manner compared to a single surge during a mass start which creates a greater impediment to the forward movement of runners. In turn, the pinch point with funnel ends creates an open space at the egress point that is less inhabited by other runners. In this way, the use of a funnel end at the egress point, following a pinch point, increases movement velocity and reduces stopping motions among crowds [22], further reducing impedance to the forward movement of runners upon race commencement. Similar to runner density data, these findings suggest using congestion-related controls (i.e., start corral design and wave starts) may reduce runner flow rate in the start corrals at large running events to create safer environments for participants by reducing the risk of runners contacting one another, slipping, tripping, and falling [27].

In addition to quantitatively measuring runner congestion during events, a mixed-method approach was used to qualitatively determine runner perceptions of congestion and its impacts on their safety and satisfaction across events with and without control strategies implemented. The survey data gathered concur with the quantitative results, showing the prevalence of congestion was higher in the 2019 event without controls than in the 2021 event with controls. More precisely, a greater proportion of runners indicated that the start corrals upon commencing the race were “somewhat” to “extremely” congested (i.e., rating of at least 3 on a 5-point Likert item) in the 2019 event (75%) compared to the 2021 event (64%). This increased runner congestion upon commencing races in the 2019 event may have filtered onto the course during races, with a significantly (*p* < 0.01) greater perceived prevalence of congestion on course while running found in the 2019 event compared to the 2021 event. In this regard, 30% of runners experienced “somewhat” to “extremely” congested conditions on course while running in the 2019 event compared to only 17% of runners in the 2021 event. In turn, the increased congestion in the 2019 event had a greater impact on the perceived safety of participants than in the 2021 event, which may be underpinned by the control strategies minimizing congestion and thus the incidence of contact with other runners, which can lead to unsafe consequences such as slips, trips, and falls [5]. Similarly, the satisfaction of a higher proportion of runners was more negatively impacted by congestion at the 2019 event compared to the 2021 event. In this regard, previous research documented that a large proportion of runners surveyed (n = 222) had experienced or witnessed bumps with other runners (87%), clashing of feet or legs (53%), or tripping and slipping at the start of the race due to congestion (38%) during running events [5]. In turn, most of these survey respondents (73%) indicated that their experiences with congestion had impacted their event satisfaction, with many (47%) highlighting that race congestion (via previous experience or feedback from others) has led to them not participating in an event [5]. Indeed, race directors acknowledge that runner congestion negatively impacts the safety and satisfaction of participants during the events they organize and emphasize the need for guidelines to be established that help them manage runner congestion [4]. Accordingly, this study provides an initial source of evidence to support the development of such guidelines regarding the use of control strategies that may subsequently improve runner congestion during running events. Appropriate use of congested-related control strategies will likely enhance runner safety and satisfaction with running events to promote continued participation. In turn, strong and consistent participation in running events across subsequent years not only enhances the viability of events [17] but may also lead to positive impacts on public health [16] and increased tourism to locations in which events are held [29]. 

### Limitations

Although this study provides novel insight regarding the impacts of control strategies on runner congestion at an Australian running event, the limitations encountered should be considered when interpreting the findings. Firstly, due to government restrictions associated with the COVID-19 pandemic, the Sunshine Coast Marathon and Running Festival was canceled in 2020. Accordingly, congestion controls were implemented at the next available opportunity in 2021 following the 2019 event. Secondly, given the real-world context of this study, the race director decided on the controls implemented in the 2021 event with input from the research team; however, given multiple control strategies were sought for implementation by the race director, the researchers could not determine the isolated impact of single control strategies on runner congestion. In this way, the use of multiple control strategies in combination is likely to be adopted at most running events and may work synergistically together in combatting runner congestion. Nevertheless, further controlled research is recommended to ascertain the effects of each control strategy on runner congestion at large running events. Thirdly, given runner congestion is heightened in the start corrals at large running events [5], only this portion of races was examined in this study. Further research is recommended for quantifying runner congestion and exploring the impact of congestion-related control strategies in other portions of races where congestion risk may be heightened (e.g., bottlenecks in course design, dramatic changes in path width, sharp turns). 

## 5. Conclusions

This study provides foundational evidence showing that the implementation of a multifaceted control approach in the form of a modified start corral design and the use of wave starts improves runner congestion, safety, and satisfaction during an Australian running event. Specifically, the peak runner densities and runner flow rates across different races were dramatically reduced with the added control strategies in place. In turn, almost double the proportion of runners surveyed indicated their safety and satisfaction were impacted by their experiences with congestion without the added control strategies. However, the discrepancy in the number of survey respondents between events (2019 event, n = 40; 2021 event, n = 172) should be considered when interpreting these qualitative results. Nevertheless, these outcomes are useful for race directors when deciding on congestion-related control strategies to implement in the running events they organize, suggesting that modified start corral designs with wave starts might offer an effective and practical (i.e., inexpensive with minimal expertise and labor required) option. In turn, concomitant improvements in runner congestion, safety, and satisfaction with the implementation of appropriate control strategies likely enhance the experience of runners during running events, increasing their likelihood of future participation [30] to provide continued benefits to runners and the community [31,32].

## Figures and Tables

**Table 1:** Survey questions distributed to runners following each running event.

Number	Question
*Characteristics*	
1	Which event did you participate in?
	a. 5 km
	b. 10 km
	c. Half-Marathon
	d. Marathon
2	Did you compete in the 2019 event?
3	How many years have you been participating in running events?
	a. Less than 1 year
	b. 1 to 3 years
	c. 3 to 5 years
	d. More than 5 years
4	Which of the following descriptions best describes you as a runner?
	a. Serious runner (I have trained in preparation for this event)
	b. Fun runner (participation is my primary goal)
*Prevalence of congestion at running event*	
5	On a scale of 1–5, with 1 being not at all congested, 2 not so congested, 3 somewhat congested, 4 very congested, and 5 extremely congested, **please rate your experience at the start corral prior to race start.**
6	On a scale of 1–5, with 1 being not at all congested, 2 not so congested, 3 somewhat congested, 4 very congested, and 5 extremely congested, **please rate your experience at the start corral as the race started and you commenced running.**
7	On a scale of 1–5, with 1 being not at all congested, 2 not so congested, 3 somewhat congested, 4 very congested, and 5 extremely congested, **please rate your experience on the course while running.**
8	On a scale of 1–5, with 1 being not at all impacted, 2 not so impacted, 3 somewhat impacted, 4 very impacted, and 5 extremely impacted, **please rate how course congestion impacted upon your event satisfaction.**
9	On a scale of 1–5, 1 being not at all unsafe, 2 not so unsafe, 3 somewhat unsafe, 4 very unsafe, and 5 extremely unsafe, **please rate how race congested impacted upon your personal safety during the race.**
10	During the race did you witness or experience any of the following (you can select more than one) a. Crush or pushing whilst you waited to run b. Bumping into other runners c. Clashing feet/legs with other runners d. Runners yelling/being aggressive about people in their way e. Inattention to other runners or race officials due to wearing headphones f. Tripping or slipping at the beginning of the race due to congestion g. Falling over whilst running due to congestion h. Being injured and requiring medical treatment due to congestion i. DNF (did not finish) due to any of the above j. Not applicable
11	Did you have any further comments regarding the congestion at this event?

*Note*: Question 2 was included only for the 2021 event.

**Table 2:** Controls implemented at the Sunshine Coast Marathon and Community Run Festival events in 2019 and 2021 to manage runner congestion.

Controls	2019 Event	2021 Event
Modified start corral design	No	Yes
Wave starts *	No	Yes
Net and gun times ^†^	Yes	Yes
Communication about controls ^‡^	No	Yes

*Note*: * wave starts involved an initial wave of elite runners selected by the race director with all subsequent waves using a self-seeding method to designate runners to waves; ^†^ net time was used for official placings and gun time was used for race winners and race records; ^‡^ conducted via a public address system explaining the pinch point in the start corral design, wave starts, and net and gun time in each race/wave.

**Table 3:** Characteristics of survey respondents participating at the Sunshine Coast Marathon and Community Run Festival events in 2019 without controls and 2021 with controls.

Question	2019 Event		2021 Event		Statistical Comparisons
	*Number*	*Percentage (%)*	*Number*	*Percentage (%)*	
*Which event did you participate in?*					
5 km	1	2	6	4	χ^2^(3) = 0.48, *p* = 0.92
10 km	7	18	37	21	
Half-Marathon	21	53	88	51	
Marathon	11	27	42	24	
*Did you compete in the 2019 event?*					
Yes	-	-	73	42	Not applicable
No	-	-	99	58	
*How many years have you been participating in running events?*					
Less than 1 year	4	10	28	16	χ^2^(3) = 3.81, *p* = 0.28
1 to 3 years	9	22	29	17	
3 to 5 years	11	28	30	17	
More than 5 years	16	40	86	50	
*Which of the following descriptions best describes you as a runner?*					
Serious runner	28	70	118	69	χ^2^(1) = 0.3, *p* = 0.86
Fun runner	12	30	54	31	

*Note*: χ^2^, Pearson’s Chi-square statistic; Question 2 was included only for the 2021 event.

**Table 4:** Survey outcomes regarding the prevalence of congestion and its impact on runner safety and satisfaction at the Sunshine Coast Marathon and Run Festival events in 2019 without controls and 2021 with controls.

Question	2019 Event		2021 Event		Statistical Comparisons
	Number	Percentage (%)	Number	Percentage (%)	
*On a scale of 1-5, please rate your experience at the start corral prior to race start.*					
Not at all congested	3	7	10	6	χ^2^(4) = 5.19, *p* = 0.27
Not so congested	7	18	50	29	2019: M, IQR = 3, 1.25
Somewhat congested	19	48	52	30	2021: M, IQR = 3, 2
Very congested	8	20	44	26	U = 3458, *p* = 0.96
Extremely congested	3	7	16	9	
*On a scale of 1-5, please rate your experience in the start corral as you commenced running.*					
Not at all congested	2	5	13	8	χ^2^(4) = 1.82, *p* = 0.77
Not so congested	8	20	49	28	2019: M, IQR = 3, 1.25
Somewhat congested	18	45	65	38	2021: M, IQR = 3, 2
Very congested	9	22	35	20	U = 3816, *p* = 0.26
Extremely congested	3	8	10	6	
*On a scale of 1-5, please rate your experience on the course while running.*					
Not at all congested	10	25	75	44	χ^2^(4) = 18.54, *p* < 0.01 *
Not so congested	18	45	68	39	2019: M, IQR = 2, 1.25
Somewhat congested	7	17	27	16	2021: M, IQR = 2, 1
Very congested	2	5	2	1	U = 4291, *p* = 0.01 *
Extremely congested	3	8	0	0	
*On a scale of 1-5 please rate how race congestion impacted upon your event satisfaction.*					
Not at all impacted	15	38	85	50	χ^2^(4) = 7.30, *p* = 0.12
Not so impacted	13	32	57	33	2019: M, IQR = 2, 2
Somewhat impacted	8	20	24	14	2021: M, IQR = 2, 1
Very impacted	4	10	4	2	U = 4017, *p* = 0.08
Extremely impacted	0	0	2	1	
*On a scale of 1-5 please rate how race congestion impacted upon your personal safety through the race*					
Not at all impacted	23	58	107	62	χ^2^(4) = 3.79, *p* = 0.44
Not so impacted	10	25	48	28	2019: M, IQR = 1, 1
Somewhat impacted	7	17	14	8	2021: M, IQR = 1, 1
Very impacted	0	0	2	2	U = 3675, *p* = 0.44
Extremely impacted	0	0	1	0	
*During the race did you witness or experience any of the following? (you can select more than one)*					
Crush or pushing whilst you waited to run	7	18	10	10	χ^2^(8) = 10.96, *p* = 0.20
Bumping into other runners	19	48	43	42	
Clashing feet/legs with other runners	5	13	17	16	
Runners yelling/being aggressive about others in their way	3	8	3	3	
Inattention to other runners or race officials due to wearing headphones	7	18	14	14	
Tripping or slipping at the beginning of the race due to congestion	4	10	2	2	
Falling over whilst running due to congestion	1	3	4	44	
Being injured and requiring medical treatment due to congestion	0	0	5	5	
DNF (did not finish) due to any of the above	0	0	5	5	
Not applicable	18	45	0	0	
*Do you have any further comments regarding your experience with congestion at this event? (themes of free-text responses)*					
Race was not congested	6	15	26	26	Not applicable
Race was congested	1	2	2	2	
Corral design	1	2	6	6	
Seeding of runners	8	22	5	5	
Wave starts	1	2	6	6	
Course design	4	10	3	3	
Race etiquette	1	2	11	11	
No further feedback/did not answer	18	45	41	41	

*Note*: χ^2^, Pearson’s Chi-square statistic; M, median; IQR, interquartile range; U, Wilcoxon–Mann–Whitney rank-sum statistic; * indicates statistically significant finding (*p* < 0.05).

## Data Availability

The data presented in this study are available on request from the corresponding author.

## Supplementary Materials

The following supporting information can be downloaded at: https://www.mdpi.com/article/10.3390/sports10090132/s1, Figure S1: An example image from video analyses with a manual count of runners shown during the 10-km race (09:01) at the 2021 Sunshine Coast Marathon and Running Festival. *Note:* Yellow lines indicate edges of designated area for counting of runners in line with telescopic poles as markers; 6 runners are identified within a 17.21 m^2^ area equating to a density of 0.35 persons per m^2^.
