# Supplementary material for: Implementation of Congestion-Related Controls Improves Runner Density, Flow Rate, Perceived Safety, and Satisfaction during an Australian Running Event
Source: Sports (Basel). 2022 Aug 31;10(9):132. doi: 10.3390/sports10090132 (PMC9500882; doi:10.3390/sports10090132)
Supplement: Supplementary file 1 [file sports-10-00132-s001.zip › sports-1824699-supplementary.pdf]

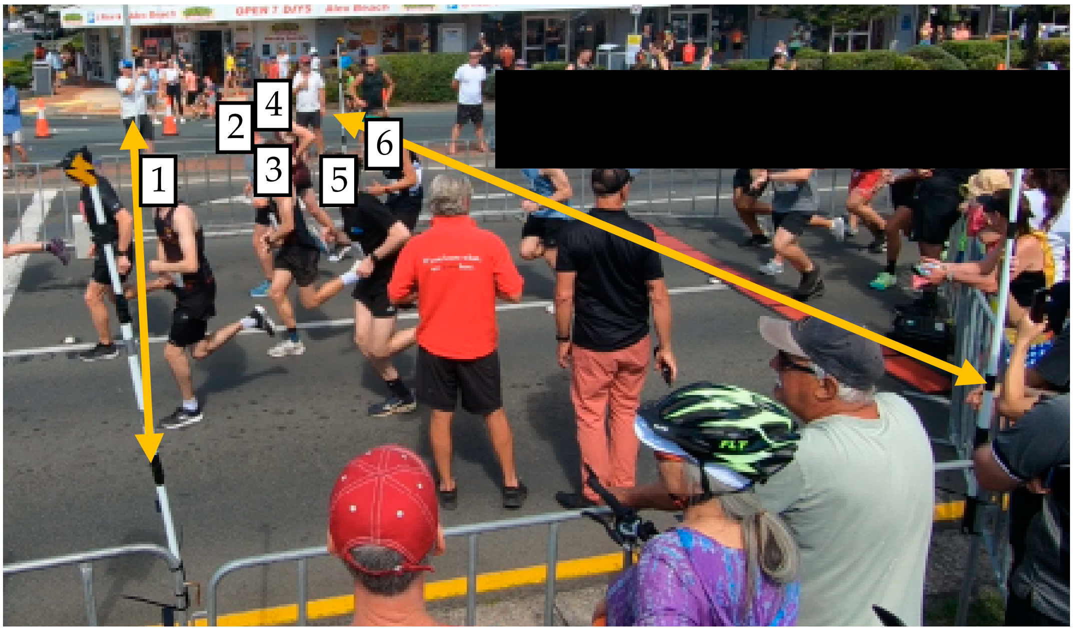

Supplementary Figure S1. An example image from video analyses with a manual count of runners shown during the 10-km race (09:01) at the 2021 Sunshine Coast Marathon and Running Festival. *Note*: Yellow lines indicate edges of designated area for counting of runners in line with telescopic poles as markers; 6 runners are identified within a 17.21 m^2 area equating to a density of 0.35 persons per m^2.
